# Supplementary material for: Single nucleosome imaging reveals loose genome chromatin networks via active RNA polymerase II
Source: J Cell Biol. 2019 Mar 1;218(5):1511–30. doi: 10.1083/jcb.201811090 (PMC6504897; doi:10.1083/jcb.201811090)
Supplement: Supplemental Materials (PDF) [file JCB_201811090_sm.pdf]

JCB

Supplemental material

Nagashima et al., https://doi.org/10.1083/jcb.201811090

Provided online are two ZIP files containing some scripts. For single-nucleosome movement analysis, the displacement and MSD of the fluorescent dots were calculated on the basis of its trajectory using a Python program. To make the heat map of chromatin dynamics, the median nucleosome movements (during 50 ms) in 3 × 3 pixels (65 nm/pixel) were plotted with a color scale from blue to red using R.

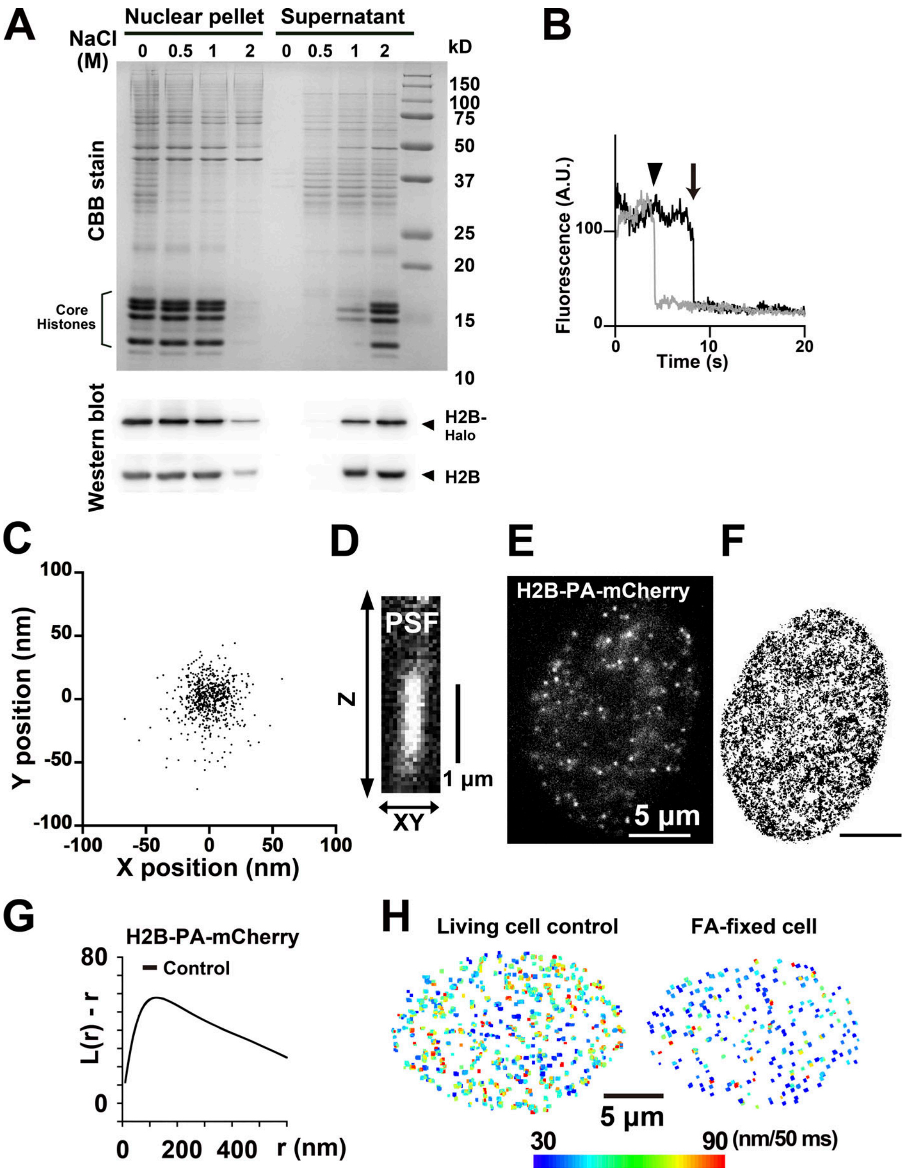

Figure S1. **Validations of H2B-Halo–labeled single nucleosomes, their position determination for accuracy, and visualization of their dynamics.** **(A)** The nuclei isolated from the RPE-1 cells expressing H2B-Halo were washed with the indicated buffers including various concentrations of NaCl. The resultant nuclear pellets (left) and supernatants (right) were analyzed by SDS-PAGE, and subsequent Coomassie brilliant blue staining (top) and Western blotting (bottom) with anti-H2B antibodies. Positions of core histones are indicated in Coomassie brilliant blue stain, and H2B-Halo and endogenous H2B are indicated with arrowheads in the Western blot. Note that H2B and H2B-Halo started to dissociate from chromatin with 1 M NaCl and were detected in the supernatant fraction, suggesting that H2B-Halo was incorporated into nucleosome structures similar to endogenous H2B. To reliably detect behavior of H2B-Halo during a salt extraction, we took a longer exposure condition for H2B-Halo detection in Western blotting than that in Fig. 1 A. **(B)** Single-step photobleaching of two representative nucleosome (H2B-Halo-TMR) dots. The vertical axis represents the fluorescence intensity of individual TMR dots. The horizontal axis is the tracking time series. The fluorescent intensity of each dot was ~120, and in the single-step photobleaching profile, the intensity dropped to around 10 (arrowhead and arrow), suggesting that each dot represents a single H2B-Halo-TMR molecule in a single nucleosome. **(C)** The position determination for accuracy of H2B-Halo-TMR. Distribution of nucleosome displacements from the centroid of their localizations in the x-y plane in the 50-ms interval. n = 10 nucleosomes in a FA-fixed cell. SDx and SDy were 13.4 nm and 15.1 nm, respectively. **(D)** Point spread function (PSF) of the observed H2B-Halo-TMR dot in a fixed RPE-1 cell. The xy-z kymograph shows that H2B-Halo-TMR can be detected in an optical layer with a thickness of ~1 µm. The vertical axis represents the depth of the optical layer (z plane; 50 nm/pixel), and the horizontal axis represents the xy plane (65 nm/pixel). **(E)** A single-nucleosome (H2B-PA-mCherry) image of the nucleus in a living RPE-1 cell. Each bright dot represents a single H2B-PA-mCherry protein. **(F)** Live-cell PALM image of histone H2B of the same cell in E. Bar, 5 µm. **(G)** The L-function plot of live RPE-1 cells expressing H2B-PA-mCherry. The L-function plot shows a curve with a peak at ~120 nm (i.e., ~240 nm in diameter). This peak size, which can provide a good approximation of the size and compaction state of the domains, is consistent with a previously reported domain size in HeLaS3 cells (Nozaki et al., 2017). Shown is a representative image among 10 PALM images. Note that PALM imaging with H2B-PA-mCherry is suitable for investigating organization of chromatin domains/nucleosome clusters in living cells. **(H)** Chromatin heat map: Visualization of nucleosome movements for 50 ms in a living (left) and FA-fixed (right) cell. The color bar represents the scale of nucleosome movement in the heat map. Small movements are shown in blue, and large movements are shown in red.

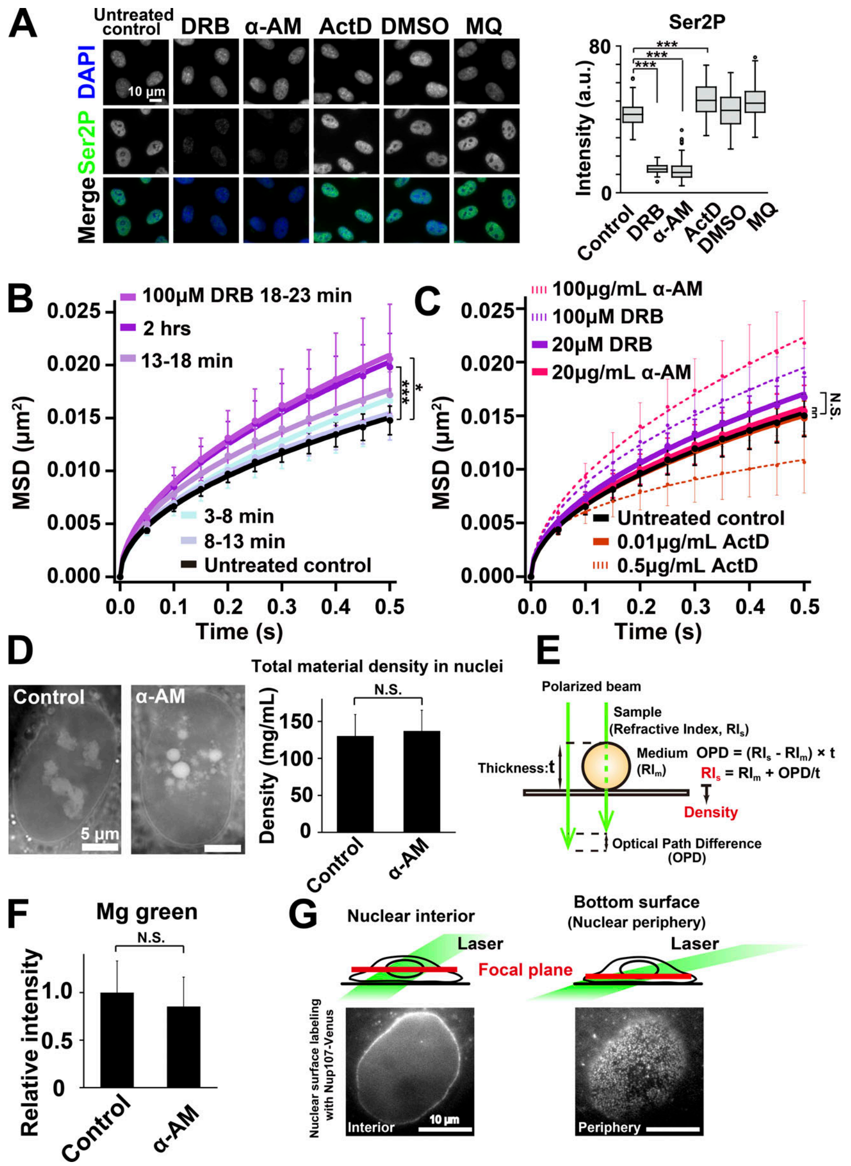

Figure S2. **RNAPII-Ser2P intensity quantification, single-nucleosome MSD with transcription inhibitor treatments, total material density imaging, free-Mg^{2+} imaging, and nuclear surface imaging. (A)** Left: RNAPII activity, which was observed by immunostaining of Ser2P, in the RPE-1 cells treated with RNAPII inhibitors (DRB, α-AM, and ActD) or without them (untreated control, DMSO, and MQ). Right: Quantification of RNAPII Ser2P signal intensity, which is an indication of RNAPII elongation, is shown as a box plot. The median intensities of Ser2P are 42.7 (n = 114) in control, 12.9 (n = 119) in DRB, 11.1 (n = 129) in α-AM, and 50.3 (n = 123) in ActD. Note that the inhibitor treatments decreased the amount of RNAPII-Ser2P, except for ActD. The Wilcoxon rank sum test shows ***, $P < 0.0001$ for control versus DRB ($P < 2.2 \times 10^{-16}$), for control versus α-AM ($P < 2.2 \times 10^{-16}$), and for control versus ActD ($P = 5.9 \times 10^{-11}$). **(B)** MSD plots (±SD among cells) of nucleosomes from 0.05 to 0.5 s in RPE-1 cells treated with DRB for the indicated shorter treatment periods or untreated (control, black). For each condition, n = 3–20 cells. Depending on the treated time with DRB, the chromatin dynamics gradually increased. *, $P < 0.05$ ($P = 0.011$) for untreated control versus 18–23 min and ***, $P < 0.0001$ ($P = 1.4 \times 10^{-7}$) for untreated control versus 2 h by the Kolmogorov–Smirnov test. **(C)** MSD plots (±SD among cells) of nucleosomes from 0.05 to 0.5 s in RPE-1 cells treated with DRB (purple), α-AM (pink), or ActD (brown) at indicated low doses or untreated (control, black). Other control data (dotted lines) were reproduced from Fig. 3 A. N.S. by the Kolmogorov–Smirnov test for untreated control versus DRB 20 µM ($P = 0.081$), for untreated control versus α-AM 20 µg/ml ($P = 0.98$), and for untreated control versus ActD 0.01 µg/ml ($P = 0.57$). **(D)** Total material densities in the nuclei of living RPE-1 cells treated with or without α-AM were measured by OI-DIC microscopy (Imai et al., 2017). Typical images of optical pass difference (OPD) map of the cells treated with (right) or without (left) α-AM are shown. Calculated total densities in the nuclei of RPE-1 cells are depicted as a bar graph with SD. The total nuclear density did not change remarkably with α-AM treatment. For each condition, n = 14–22 cells. N.S. ($P = 0.50$) by Student's t test. **(E)** A procedure for estimating sample (depicted as a sphere) RI. OI-DIC microscopy can computationally quantify OPDs at each spatial point. For details, see Materials and methods and Imai et al. (2017). **(F)** Relative free Mg^{2+} concentrations in RPE-1 cells treated with (right) or without (left) α-AM were measured by the free Mg^{2+} indicator, Mg-green, and were shown as bar graph with SD. For each condition, n = 33–42 cells. The free Mg^{2+} concentration also did not change remarkably with α-AM treatment. N.S. ($P = 0.052$) by Student's t test. **(G)** A schematic representation for nuclear interior and nuclear surface (periphery) imaging. A focal plane (red) at nuclear interior (left) or periphery (right) was selected by changing the angle of laser illumination (green). Note that the two different focal planes were precisely verified by nuclear surface labeling with a nuclear pore component NUP107-Venus (Maeshima et al., 2010b): Almost uniform distributions of NUP107-Venus were observed in the nuclear periphery condition (right), while the nuclear interior condition showed a rim of NUP107-Venus (left).

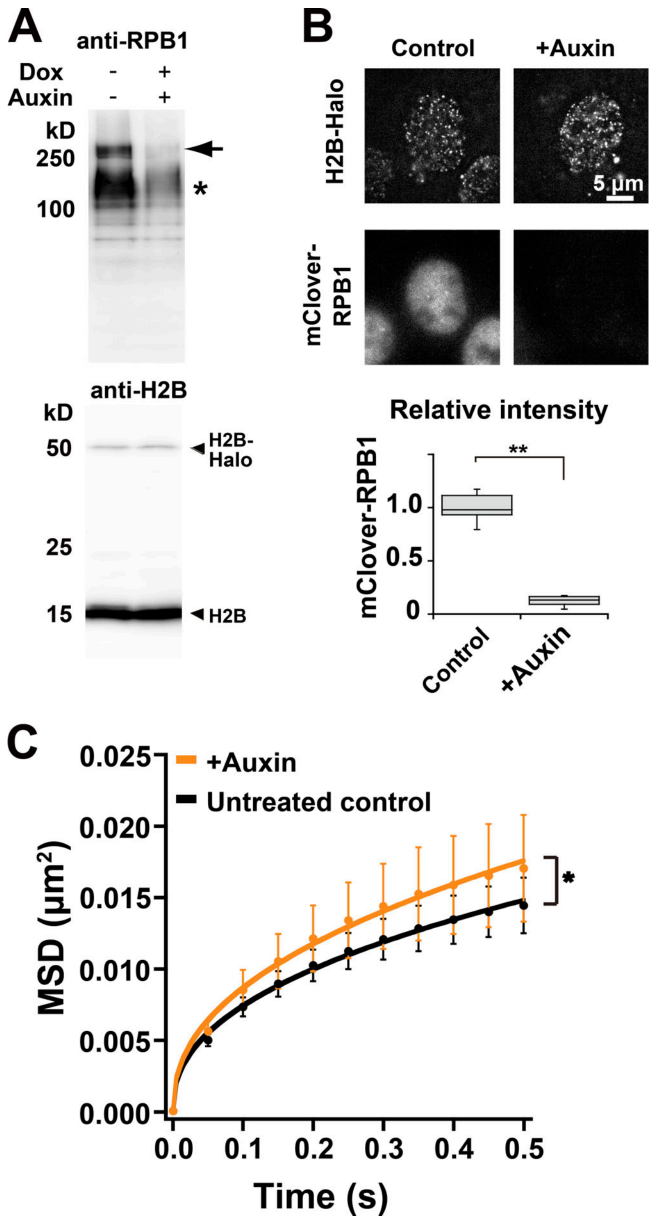

Figure S3. **Rapid degradation of RNAPII in HCT116 cells and increased the chromatin dynamics. (A)** Top: Rapid RNAPII degradation in human colorectal cancer HCT116 cells upon auxin addition was confirmed by Western blotting with anti-RPB1 CTD antibody (arrow). In the HCT116 cells, the anti-RPB1 antibody somehow produced an additional nonspecific band in Western blotting (marked with asterisk). This cell line has RPB1 tagged with mAID-mClover (mAC-RPB1) in both alleles and tetracycline-inducible Tet-OsTIR1 at the AAVS1 locus. Bottom: The same sample sets as the top blot were probed with anti-H2B antibody, showing H2B-Halo and endogenous H2B. **(B)** Confirmation of RNAPII degradation in HCT116 cells after the auxin treatment by imaging of mClover-RPB1. Fluorescent images of mClover-RPB1 (bottom) and H2B-Halo-TMR (bottom) in the same live HCT116 cells before (left) and after (right) treatment with auxin for 2 h are shown. The relative intensities of mClover-RPB1 normalized to the untreated control are shown as box plots. *n* = 11 cells for +Auxin, and 12 cells for control. **, $P < 0.0001$ ($P < 1.5 \times 10^{-6}$) by the Wilcoxon rank sum test. **(C)** MSD plots (±SD among cells) of nucleosomes in HCT116 cells before (control, black) and after auxin addition (+Auxin, orange). *n* = 27 cells in the untreated control; *n* = 37 cells with auxin treatment. *, $P < 0.05$ ($P = 0.012$) by the Kolmogorov–Smirnov test. Note that the prompt degradation of RNAPII increased the chromatin dynamics.

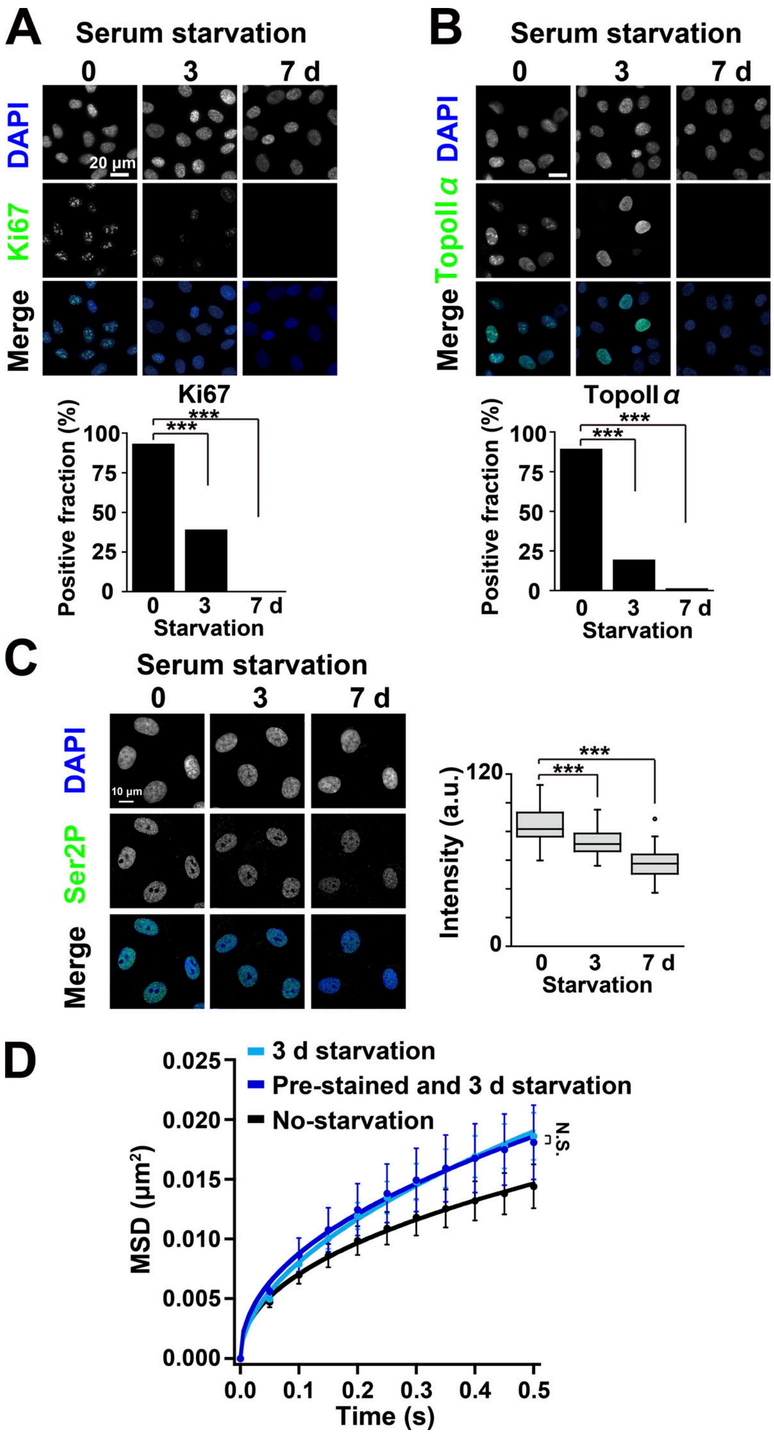

Figure S4. **Immunostaining of Ki67, TopoIIα, and RNAPII-Ser2P and nucleosome dynamics in serum-starved cells. (A and B)** Verification of G0 resting state of RPE-1 cells by immunostaining with proliferation markers Ki67 (A) and TopoIIα (B). Bars, 20 µm. Ki67 and TopoIIα positive fractions are shown as a bar graph (bottom in A and B). The Ki67-positive cells were 93% in the 76 nonstarved cells (0 d), 39% in the 107 cells starved for 3 d, and 0% in the 111 cells starved for 7 d. ***, $P < 0.0001$ by Fisher's exact test for 0 d versus 3 d ($P = 5.0 \times 10^{-16}$) and for 0 d versus 7 d ($P < 2.2 \times 10^{-16}$). The TopoIIα-positive cells were 90% in the 67 nonstarved cells (0 d), 20% in the 97 cells starved for 3 d, and 1.5% in the 67 cells starved for 7 d. ***, $P < 0.0001$ by Fisher's exact test for 0 d versus 3 d ($P < 2.2 \times 10^{-16}$) and for 0 d versus 7 d ($P < 2.2 \times 10^{-16}$). **(C)** Left: Verification of RNAPII activity of RPE-1 cells without (0 d) or with the serum starvation for 3 d and 7 d by immunostaining of Ser2P of the RPB1 CTD in RNAPII. Right: Quantifications of RNAPII Ser2P signal intensity are shown as box plots. The median intensities of Ser2P are 81.7 (n = 41) in 0-d starvation, 71.3 (n = 42) in 3-d starvation, and 57.6 (n = 39) in 7-d starvation. RNAPII activity decreased after serum starvation. ***, $P < 0.0001$ by the Wilcoxon rank sum test for 0 d versus 3 d ($P = 9.5 \times 10^{-12}$) and for 0 d versus 7 d ($P < 2.2 \times 10^{-16}$). **(D)** MSD plots (±SD among cells) of nucleosomes in RPE-1 cells without starvation (black) and with 3-d serum starvation (light blue) and TMR-prestained nucleosomes with the 3-d starvation (prestained, dark blue) to exclude the possibility that the starvation-induced chromatin change might affect TMR-labeling. For each condition, n = 14–27 cells. Note that the chromatin dynamics were unchanged regardless of when the nucleosomes were labeled with TMR dye. N.S. ($P = 0.98$) by the Kolmogorov–Smirnov test.

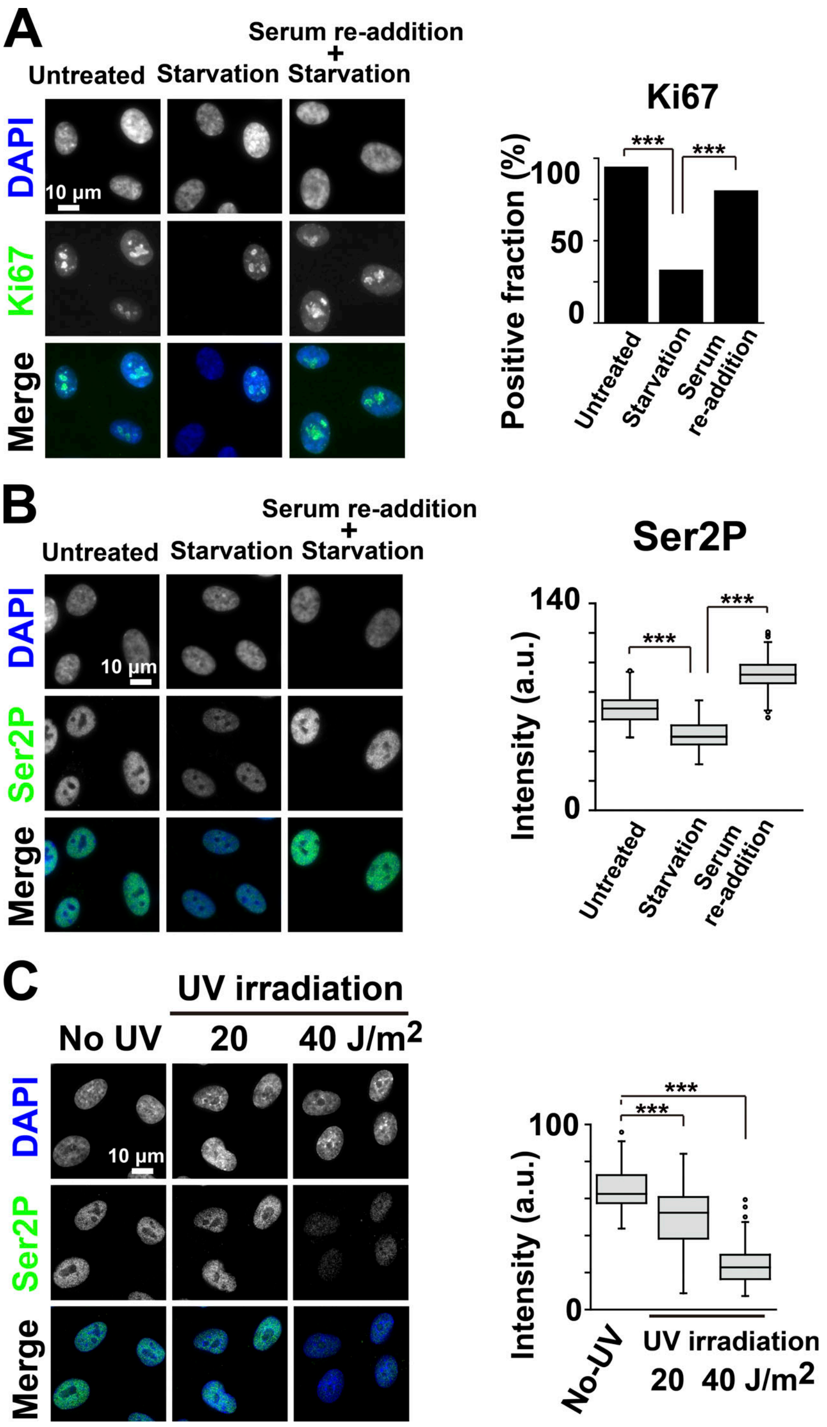

Figure S5. **Immunostaining of Ki67 and RNAPII-Ser2P in serum-restored cells and RNAPII-Ser2P immunostaining in UV-irradiated cells. (A)** Left: Verification of proliferation state re-entered from G0 phase by immunostaining of Ki67. Right: Positive fractions of Ki67 signal are shown as a bar graph. The values are 95% (*n* = 101 cells) in the cells without starvation, 32% (*n* = 111 cells) in those with 3-d starvation, and 81% (*n* = 67 cells) in the cells with serum re-addition after 3-d starvation. ***, $P < 0.0001$ by Fisher's exact test for untreated versus starvation ($P < 2.2 \times 10^{-16}$) and for starvation versus serum re-addition ($P = 2.9 \times 10^{-10}$). **(B)** Left: Verification of RNAPII activity by immunostaining of Ser2P in the RPE-1 cells without or with 3-d serum starvation and serum re-addition following the 3-d starvation. Right: Quantifications of RNAPII Ser2P signal intensity are shown as box plots. The median intensities of Ser2P are 68.7 (*n* = 105 cells) in the cells without starvation, 49.8 (*n* = 96 cells) in 3-d starvation, and 91.7 (*n* = 67 cells) in serum re-addition. Note that RNAPII-Ser2P decreased in the G0 phase and was restored upon the re-entering the cell cycle after the serum re-addition. ***, $P < 0.0001$ by the Wilcoxon rank sum test for untreated versus starvation ($P < 2.2 \times 10^{-16}$) and for starvation versus serum re-addition ($P < 2.2 \times 10^{-16}$). **(C)** Left: Verification of RNAPII activity of RPE-1 cells before (no UV) or after 20- or 40-J/m^2 UV irradiation by immunostaining of Ser2P in RNAPII. Right: Quantifications of RNAPII Ser2P signal intensity are shown as box plots. The median intensities of Ser2P are 62.5 (*n* = 114 cells) in control, 52.3 (*n* = 94 cells) in 20 J/m^2, and 22.8 (*n* = 89 cells) in 40 J/m^2. RNAPII activity decreased after the UV irradiation. ***, $P < 0.0001$ by the Wilcoxon rank sum test for no UV versus 20 J/m^2 ($P = 9.5 \times 10^{-12}$) and for no UV versus 40 J/m^2 ($P < 2.2 \times 10^{-16}$).

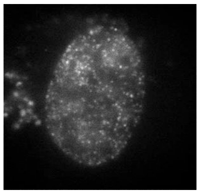

Video 1. **Movie data (50 ms/frame) of single nucleosomes labeled with TMR in a living RPE-1 cell.** Note that, after background subtraction, clear, well-separated dots and their movements were visualized with a single-step photobleaching profile (see Fig. S1 B), suggesting that each dot represents a single H2B-Halo-TMR molecule in a single nucleosome.

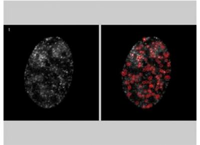

Video 2. **A tracking example of Video 1 data.** The tracked data by u-track (MATLAB package; Jaqaman et al., 2008) was overlaid on the original movie and marked with red circles (right).

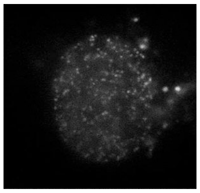

Video 3. **Movie data (50 ms/frame) of single nucleosomes labeled with TMR in the living RPE-1 cell treated with α-AM at 100 µg/ml for 2 h.** Note that greater movements of the dots are observed than those in Video 1, suggesting the local chromatin dynamics increased upon transcription inhibition.

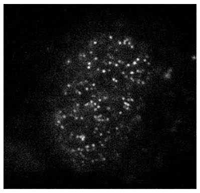

Video 4. **Movie data (50 ms/frame) of single nucleosomes labeled with TMR in the nuclear periphery (nuclear bottom surface) of a living RPE-1 cell.** Note that much less movement of the dots is observed than that in Video 1, suggesting the local chromatin dynamics are suppressed in the heterochromatic regions.

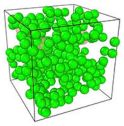

Video 5. **Movie of computational modeling for 1.5 s of movement of four chromatin chains with domains (green spheres) and four hubs (faint pink spheres, P-TEFb clusters) in the absence of the glues (RNAPII-Ser5P).** Coarse-grained 100-kb chromatin domains (green) connected by invisible springs and hubs (pink) are shown with spheres. Note that for better visualization of their spatial distributions and movements green and pink spheres are drawn with a radius of 90 and 180 nm, respectively, although the SDs of their density distributions assumed in the model are ~70 and 210 nm, respectively. The movie file was created by using the software OVITO (Stukowski, 2010).

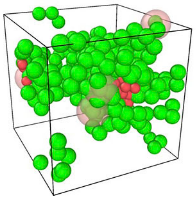

Video 6. **Movie of computational modeling for 1.5 s of movement of four chromatin chains with domains (green spheres), four hubs (faint pink spheres, P-TEFb clusters), and 64 glues (small red spheres, RNAPII-Ser5P).** Coarse-grained 100-kb chromatin domains (green) connected by invisible springs, hubs (pink) and glues (red) are shown with spheres. Note that for better visualization of their spatial distributions and movements green, red, and pink spheres are drawn with radius, 90, 60, and 180 nm, respectively, although the SDs of their density distributions assumed in the model are ~70, 70, and 210 nm, respectively. The movie file was created by using the software OVITO (Stukowski, 2010).

References

Imai, R., T. Nozaki, T. Tani, K. Kaizu, K. Hibino, S. Ide, S. Tamura, K. Takahashi, M. Shribak, and K. Maeshima. 2017. Density imaging of heterochromatin in live cells using orientation-independent-DIC microscopy. *Mol. Biol. Cell.* 28:3349–3359. https://doi.org/10.1091/mbc.e17-06-0359

Jaqaman, K., D. Loerke, M. Mettlen, H. Kuwata, S. Grinstein, S.L. Schmid, and G. Danuser. 2008. Robust single-particle tracking in live-cell time-lapse sequences. *Nat. Methods.* 5:695–702. https://doi.org/10.1038/nmeth.1237

Maeshima, K., H. Iino, S. Hihara, T. Funakoshi, A. Watanabe, M. Nishimura, R. Nakatomi, K. Yahata, F. Imamoto, T. Hashikawa, et al. 2010b. Nuclear pore formation but not nuclear growth is governed by cyclin-dependent kinases (Cdks) during interphase. *Nat. Struct. Mol. Biol.* 17:1065–1071. https://doi.org/10.1038/nsmb.1878

Nozaki, T., R. Imai, M. Tanbo, R. Nagashima, S. Tamura, T. Tani, Y. Joti, M. Tomita, K. Hibino, M.T. Kanemaki, et al. 2017. Dynamic Organization of Chromatin Domains Revealed by Super-Resolution Live-Cell Imaging. *Mol. Cell.* 67:282–293.e7. https://doi.org/10.1016/j.molcel.2017.06.018

Stukowski, A. 2010. Visualization and analysis of atomistic simulation data with OVITO–the Open Visualization Tool. *Model. Simul. Mater. Sci. Eng.* 18:015012. https://doi.org/10.1088/0965-0393/18/1/015012
